# Pharmacological treatment with galectin-1 protects against renal ischaemia-reperfusion injury

**DOI:** 10.1038/s41598-018-27907-y

**Published:** 2018-06-22

**Authors:** Carla P. Carlos, Analice A. Silva, Cristiane D. Gil, Sonia M. Oliani

**Affiliations:** 1 Department of Biology, Instituto de Biociências, Letras e Ciências Exatas, Sao Paulo State University, UNESP, São José do Rio Preto, SP Brazil; 2 Department of Medicine, FACERES School of Medicine, São José do Rio Preto, SP Brazil; 3 Department of Morphology and Genetics, Federal University of Sao Paulo, UNIFESP, São Paulo, SP Brazil

## Abstract

Galectin-1 protein (GAL-1) has important anti-inflammatory properties, but related pharmacologic approaches to effectively treat or prevent renal ischaemia and reperfusion injury are highly limited. Here, we investigated the effect of GAL-1 in a renal ischaemia-reperfusion injury rat model and an *in vitro* hypoxia-reoxygenation model with a proximal renal tubular epithelial cell line. *In vivo*, pretreatment with GAL-1 attenuated the renal parameters changed by ischaemia-reperfusion/hypoxia-reoxygenation, with recovery of renal function, protecting against influx of leukocytes, cell death and oxidative stress. Ischaemia-reperfusion/hypoxia-reoxygenation was also associated with increased renal endogenous expression of GAL-1 and intercellular adhesion molecule 1 (ICAM-1) plus augmented levels of proinflammatory cytokines IL-1β, TNF-α and MCP-1 and decreased anti-inflammatory IL-10 in urine, all of which were abrogated by GAL-1 treatment. *In vitro* studies demonstrated renal tubular epithelial cells as an important source of GAL-1 during hypoxia-reoxygenation and confirmed the protective effects of exogenous GAL-1 through downregulation of proinflammatory cytokine release by proximal renal tubular epithelial cells. Collectively, our findings confirm the important anti-inflammatory role of GAL-1 in kidney ischaemia and reperfusion injury and indicate its promising use as a therapeutic approach.

## Introduction

Ischaemia-reperfusion (IR) occurs during procedures involved in the transplantation of organs and the damage caused by hypoxic IR in tubular cells represents the first event of acute renal failure^1,2^. This condition is associated with high rate of morbidity and mortality once blood reperfusion induces the production of reactive oxygen species and, consequently, causes inflammatory response, cell death and organ failure^1–5^. Thus, understanding the cellular and molecular pathways involved in IR condition is fundamental to determine therapeutic tools to avoid the damage involved in renal transplantation.

An increasing amount of experimental evidence has demonstrated the involvement of galectins as mediators of inflammation acting at the beginning, amplification or resolution of this process, mainly galectin-1 (GAL-1). Galectins are lectin family members defined by their affinity for β-galactoside carbohydrates and their shared consensus amino acid sequences in the carbohydrate recognition domain (CRD). They are widely expressed in various tissues and organs, showing highest expression in the immune system^6^.

GAL-1 is a 14.5 kDa protein that modulates cellular signalling, proliferation and survival and plays critical roles in the control of acute and chronic inflammation and neovascularization^6–8^. This lectin alters the secretion of cytokines, reducing levels of IL-2, IL-12, interferon gamma (IFN-γ) and tumour necrosis factor (TNF-α) and increasing IL-5 and IL-10^6,7,9,10^. Additionally, exogenous GAL-1 can also negatively regulate neutrophil influx into the peritoneal cavity^11–13^, an effect associated with alteration of the levels of adhesion molecules L-selectin and ß2-integrin^13–15^. Thus, GAL-1 represents a biological mediator that has a therapeutic potential for inflammatory diseases and is a possible target for IR therapy.

Given the acute renal failure is associated with increased risk of mortality and that the immune regulatory functions of GAL-1 in renal injury processes are poorly understood, we aimed to determine the mechanism of action of this lectin in an *in vivo* and an *in vitro* model of renal IR injury.

## Results

### GAL-1 ameliorates the renal lesion and function induced by IR

Animals subjected to the IR condition showed a marked damage in the renal function (Table 1). IR group exhibited increased blood urea nitrogen (BUN) levels, plasma creatinine levels and excretion of sodium and potassium, as wells as reduced creatinine clearance and urinary volume. Administration of GAL-1 or dexamethasone (DEXA) abrogated the effects of IR on kidney function. Animals from all experimental groups lost weight (Table 1).

**Table 1:** Effect of GAL-1 treatment on weight loss and renal function induced by IR injury.

Parameter	Group			
	SHAM	IR	IR + DEXA	IR + GAL-1
Weight loss (g)	−18.3 ± 10.6	−11.7 ± 32.1	−28.8 ± 18.2	−38.0 ± 32.2
Plasma creatinine (mg/dL)	0.60 ± 0.12	2.47 ± 2.37^++^	0.65 ± 0.12	0.50 ± 0.07
Creatinine clearance (mL/min/100 g)	0.29 ± 0.13	0.16 ± 0.18^+^	0.35 ± 0.07	0.44 ± 0.17
Urinary output (mL/24 h)	12.2 ± 2.4	7.3 ± 3.0**	14.3 ± 1.9	11.8 ± 3.2
Sodium excretion fraction (%)	0.22 ± 0.04	0.54 ± 0.20***	0.19 ± 0.05	0.21 ± 0.05
Potassium excretion fraction (%)	39.4 ± 14.7	65.6 ± 32.0*	29.0 ± 7.0	32.8 ± 8.9
BUN (mg/dL)	18.0 ± 2.7	46.0 ± 24.9^†^	27.0 ± 6.7	21.6 ± 2.8^+^
TBARS (mMol/L)	112.6 ± 19.1^$^	155.5 ± 24.9	207.5 ± 19.0	174.7 ± 12.6
KIM-1 (pg/mL)	213.5 ± 76.2	982.9 ± 96.7^#^	943.9 ± 117.0^#^	814.9 ± 43.7

^a^Data represent the mean ± SD. N = 6 rats.

^b^The IR group showed a reduction in renal function in comparison with the SHAM, DEXA or GAL-1-treated IR groups, as detected by the analysis of different renal parameters. No difference in weight loss was observed among groups (p > 0.05). One-way ANOVA or Kruskal-Wallis test.

^+^p < 0.05 and ^++^p < 0.01, IR vs IR + GAL-1.

^*^p < 0.05, **p < 0.01 and ***p < 0.001, IR vs all groups.

^†^p < 0.05, IR vs SHAM.

^$^p < 0.05, SHAM vs all groups.

^#^p < 0.05, IR and IR + DEXA vs SHAM.

**Figure 1:**
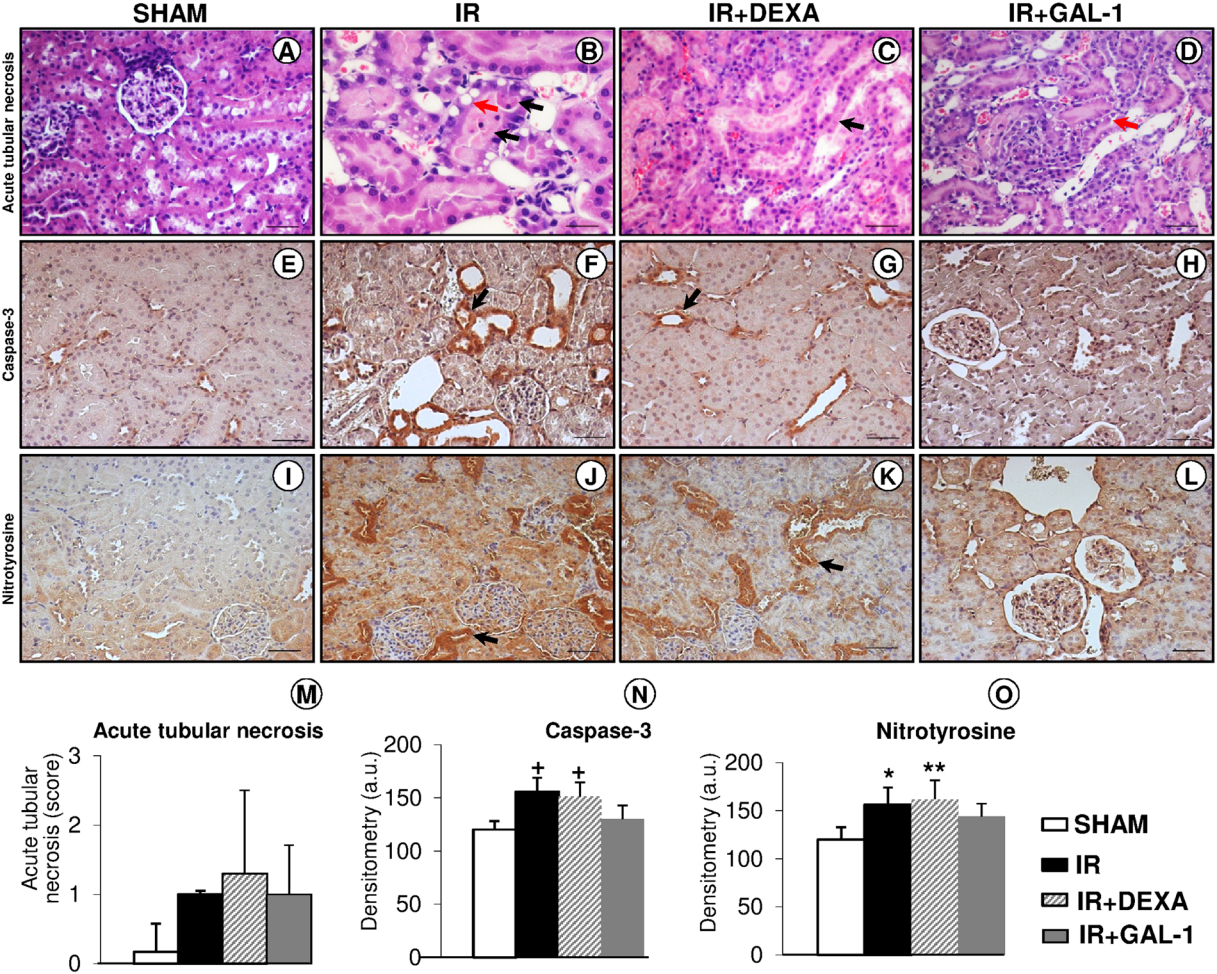
Acute tubular necrosis, apoptosis and oxidative stress. (**A–D**) Vacuolized necrotic tubular cells (red arrows), pyknosis, karyolysis, karyorrhexis (black arrows), and casts of tubular light chains were observed in IR groups. Stain: haematoxylin-eosin. (**E–L**) The ischaemia-reperfusion (IR) and IR + DEXA groups showed enhanced expression of caspase-3 and nitrotyrosine (arrows), compared with the SHAM and IR + GAL-1 groups. Counterstain: haematoxylin. Bars: 75 μm (**A**, **C**–**L**); 50 μm (**B**). (**M**) Semi-quantitative score of acute tubular necrosis. (**N/O)** Densitometric analysis of caspase-3 and nitrotyrosine expression (arbitrary units, a.u.). Data represent the mean ± SD, N = 6/group. ^+^p < 0.05, IR and IR + DEXA vs SHAM and IR + GAL-1. *p < 0.05 and **p < 0.01 vs SHAM. One-way ANOVA or Kruskal-Wallis test.

DEXA and GAL-1-treated IR groups presented mild to moderate tissue injury of the juxtamedullary region. Acute tubular necrosis was characterized as previously described^16^, i.e., presence of vacuolized necrotic tubular cells and with intense acidophilia, pyknosis, karyolysis, karyorrhexis, casts of tubular light chains, loss of the tubular brush border and presence of inflammatory cells (Fig. 1A–D). Analysis of acute tubular necrosis score from all experimental groups showed no significant differences (Fig. 1M). However, the IR and IR + DEXA groups but not the IR + GAL-1 group presented elevated levels of KIM-1 in the urine, compared to the SHAM group (Table 1).

In addition, the IR and IR + DEXA groups exhibited enhanced cell death in the juxtamedullary region, as detected by expression of caspase-3, in comparison to the SHAM and IR + GAL-1 groups (Fig. 1E–H,N).

### GAL-1 reduces oxidative stress in kidney

Analysis indicated that the IR and IR + DEXA groups also exhibit elevated kidney oxidative stress by enhanced nitrotyrosine expression in the juxtamedullary region compared with the SHAM group (Fig. 1I–K,O). GAL-1 treatment reversed this effect (Fig. 1L,O). However, all groups submitted to IR (treated or not) presented elevated plasma TBARS (thiobarbituric-acid-reactive substances) levels compared with the SHAM group (Table 1).

### GAL-1 reduces neutrophil and macrophage influx to the juxtamedullary kidney through the modulation of adhesion molecules

**Figure 2:**
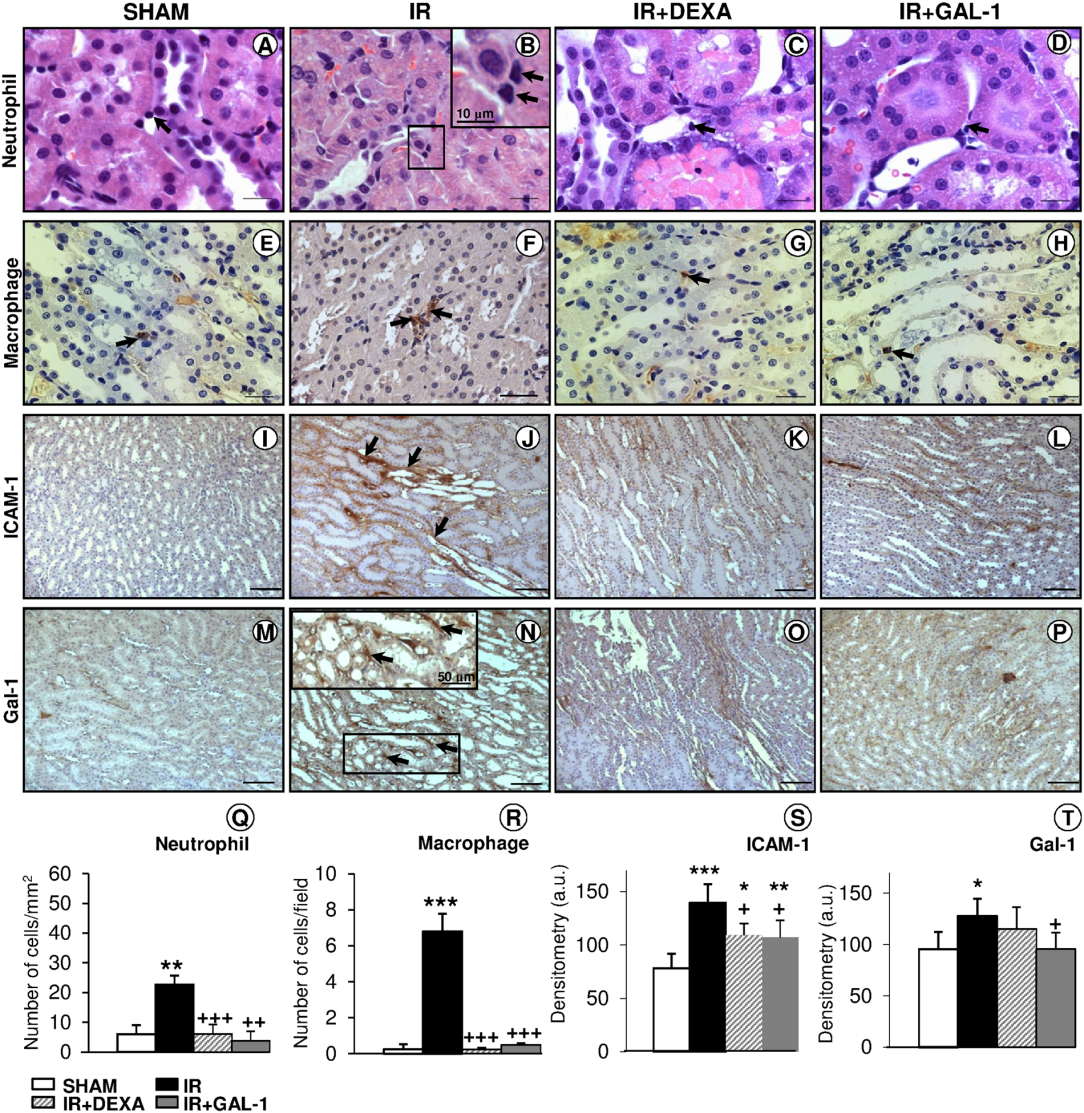
Inflammatory cell influx and ICAM-1/GAL-1 expression in the juxtamedullary region. (**A–H**) The ischaemia-reperfusion (IR) group showed intense neutrophil and macrophage infiltration in the juxtamedullary region (arrows; B and F, respectively) compared to the SHAM and DEXA- or GAL-1-treated groups. Details of two neutrophils in Figure B (inset). (**I–P**) The IR group exhibited enhanced expression of ICAM-1 and GAL-1 in the region with tubular necrosis (arrows) compared with all groups. Detail of the interstitial GAL-1 immunostaining in Figure N (inset). (**A–D**) Stain: haematoxylin-eosin. (**E–P**) Counterstain: haematoxylin. Bars: 20 μm (**A**–**D**); 50 μm (**E**–**H**); 100 μm (**I**–**P**). (**Q–R**) Quantitative analyses of neutrophils and macrophages. Data represent the mean ± SD of the cell number per mm^2^ or field. (**S–T**) Densitometric analysis of ICAM-1 and GAL-1 expression. Data (arbitrary units, a.u.) represent the mean ± SD of protein immunoreactivity. (N = 6/group). *p < 0.05, **p < 0.01 and ***p < 0.001, vs SHAM. ^+^p < 0.05, ^++^p < 0.01 and ^+++^p < 0.001 vs IR, one-way ANOVA.

After IR, juxtamedullary region showed an intense influx of neutrophils and macrophages compared to the SHAM group (Fig. 2A–B,E–F and Q–R). In contrast, GAL-1 and DEXA treatments reversed this effect, showing diminished inflammatory cell numbers compared to the non-treated IR group (Fig. 2B–D,F–H and Q–R).

**Figure 3:**
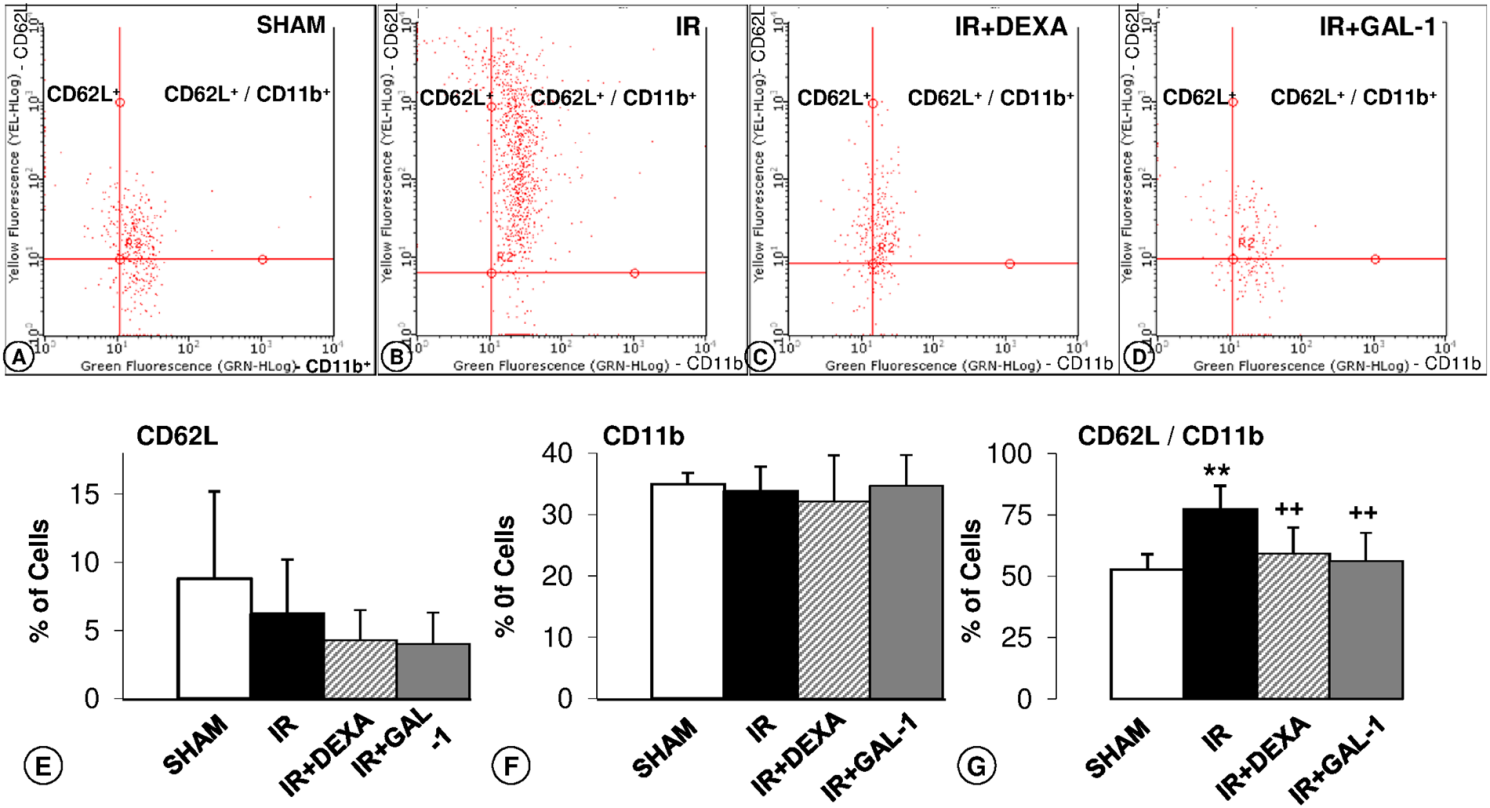
GAL-1 regulates blood leukocyte recruitment. (**A–D**) Representative dot plots of leukocytes (each point represents a cell) immunostained for adhesion molecules CD62L (conjugated with PE) and CD11b (conjugated with FITC). Groups: (**A)** SHAM, (**B**) ischaemia-reperfusion (IR), (**C)** IR treated with dexamethasone (DEXA), or (**D**) GAL-1, 30 min before IR. (**E–G**) Percentage of CD62L^+^, CD11b^+^ or double labelled to CD62L^+^/CD11b^+^. The percentage of CD62L^+^/CD11b^+^leukocytes increased in the IR group. These alterations were reversed with DEXA or GAL-1 treatments. (**F**) There was no significant difference in CD62L^+^ and CD11b^+^ expression among the studied groups. Data represent the median ± SD of the percentage of cells. **p < 0.01, IR vs SHAM. ^++^p < 0.01 and ^+++^p < 0.001 vs IR, one-way ANOVA test, N = 6/group.

To better understand this anti-migratory effect of GAL-1 under IR, we investigated blood leukocyte recruitment under different experimental conditions via flow cytometry (Fig. 3). The analysis of IR dot plot graphs demonstrated a high incidence of CD62L/CD11b-positive cells (Fig. 3B) compared to the other experimental groups (Fig. 3A,C and D). Analysis of the percentages of CD62L/CD11b-positive cells confirmed this observation (Fig. 3G), and no difference was detected for only positive CD62L (Fig. 3E) or CD11b cells (Fig. 3F) among the studied groups.

Additionally, renal IR injury enhanced ICAM-1 and GAL-1 expression in the interstitial tissue of necrosed tubules of the juxtamedullary region compared to the SHAM group (Fig. 2I–J,M–N and S–T). Pre-treatment with GAL-1 reduced its endogenous expression (Fig. 2P and T) and ICAM-1 levels (Fig. 2L and S) in the juxtamedullary region in comparison to the untreated IR and SHAM groups. No change in the interstitial expression of GAL-1 proteins was detected in the DEXA-treated group (Fig. 2O and T), but ICAM-1 expression was reduced in this group (Fig. 2K and S).

### GAL-1 and DEXA treatments protect HK-2 cells against HR-induced cytotoxicity

**Figure 4:**
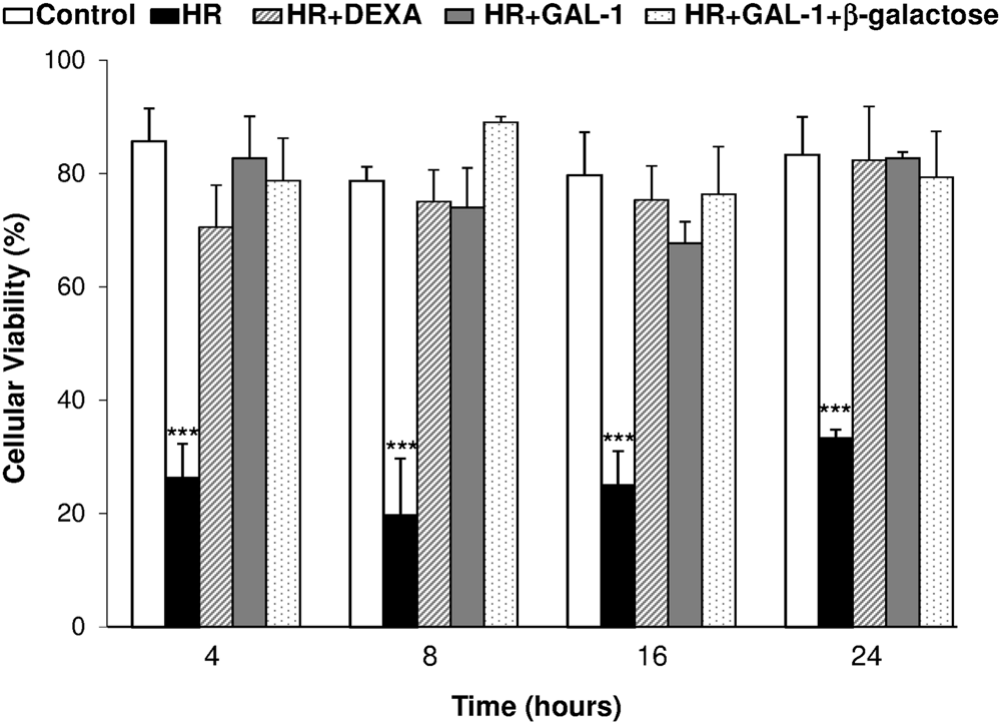
Effect of GAL-1 treatment on HK-2 cell viability under hypoxia-reoxygenation (HR). Control cells, HR, HR treated with DEXA 1 mM/mL, GAL-1 4 mg/mL or GAL-1 + β-Galactose 100 mM/mL, 30 min before HR. Histogram showing lower cellular viability at 4, 8, 16 or 24 h after HR, compared to the control. The cellular viability did not differ among control and treated cells at the different times studied. Data are shown as the mean ± SD of three experiments. ***p < 0.001, vs all groups, one-way ANOVA test, N = 3/group.

The viability of the cells was investigated in HK-2 control cells exposed to hypoxia and reoxygenation (HR) and HR incubated with GAL-1 (4 µg/mL) or DEXA (1 µM) at experimental times 4, 8, 16 and 24 h after HR. At all times assessed, the viability of the HK-2 cells submitted to HR was significantly lower than that of the control cells (Fig. 4) and was abrogated after GAL-1 or DEXA treatments. This effect could be observed by the presence of dead cells not adhered after 24 h of the HR procedure.

### Release of pro- and anti-inflammatory cytokines is regulated by GAL-1 treatment in ***in vivo*** and ***in vitro*** models of IR

**Figure 5:**
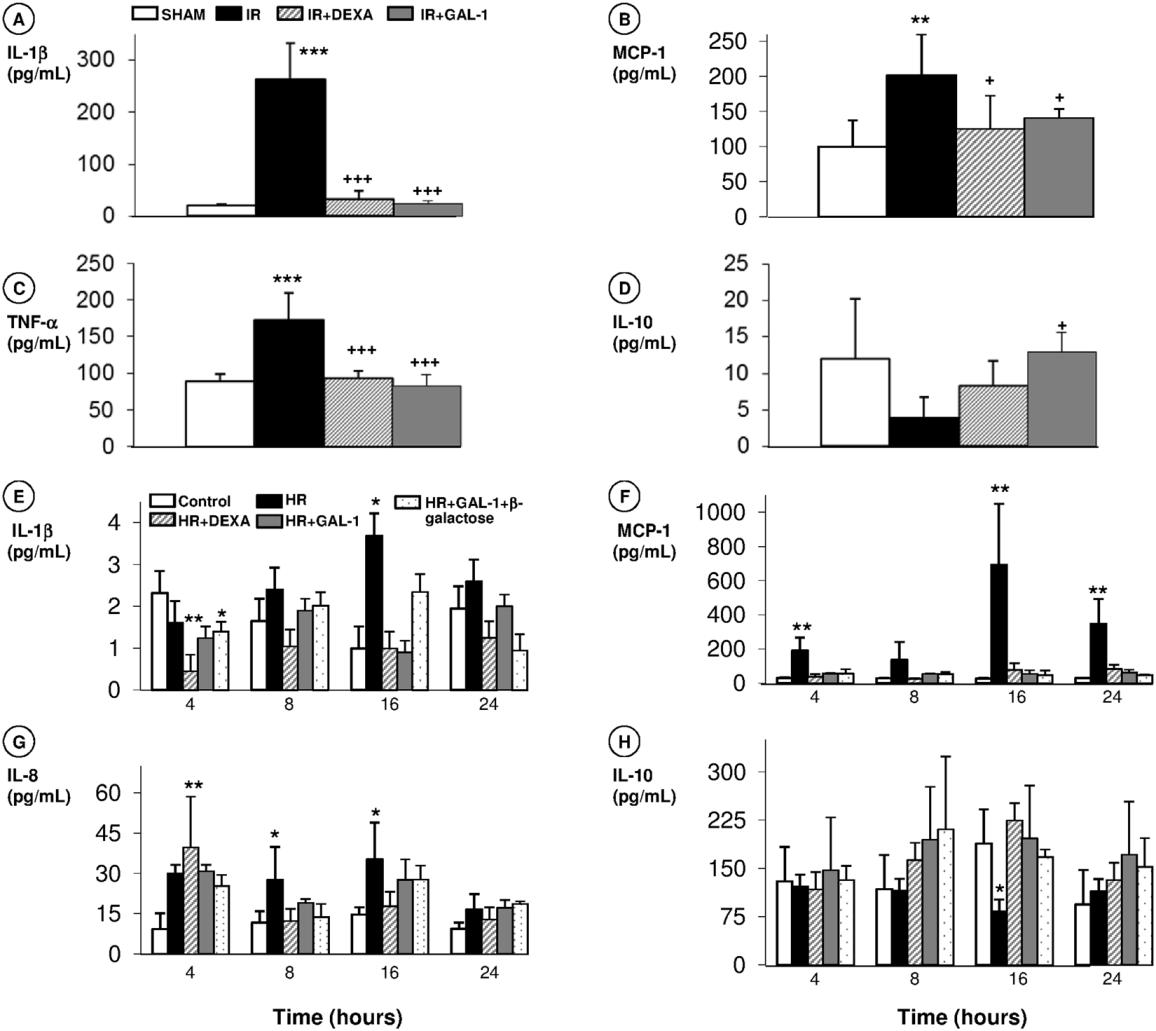
Effect of GAL-1 treatment on cytokine release in the *in vivo* and *in vitro* IR models. **(A–C)** *In vivo*, the IR group exhibited elevated urine levels of (**A**) IL-1β, (**B**) MCP-1, and (**C**) TNF-α, while the DEXA and GAL-1-treated groups showed reduced IL-1β. (**D**) GAL-1 treatment induced elevated IL-10 levels in the urine compared to the levels in the untreated IR group. Data are expressed as the mean ± SD (pg/mL) from the urine (N = 6/group). **p < 0.01 and ***p < 0.01 vs SHAM. ^+^p < 0.05 and ^+++^p < 0.001 vs IR. (**E–H)** *In vitro*, supernatant levels of (**E)** IL-1β, (**F)** MCP-1, (**G)** IL-8, and (**H)** IL-10, under control, HR, HR treated with DEXA or GAL-1 (with or without β-galactose) conditions at 4, 8, 16 or 24 h. HR increased the IL-1β levels at 16 h; the MCP-1 levels at 4, 16 and 24 h; and the IL-8 levels at 8 and 16 h and reduced the IL-10 levels at 16 h. DEXA elevated IL-8 release at 4 h and reduced IL-1β at 4 h compared to the control. These alterations were reversed with GAL-1 or GAL-1 + β-galactose treatments. Data are expressed as the mean ± SD (pg/mL) from the HK-2 cell supernatants (N = 3/group). *p < 0.05 and **p < 0.01, vs control, one-way ANOVA or Kruskal-Wallis test.

*In vivo*, renal IR markedly increased the urine levels of IL-1β, MCP-1 and TNF-α (Fig. 5A–C) compared to the levels in SHAM animals. Both pharmacological treatments, GAL-1 and DEXA, decreased these levels in the urine after the IR condition (Fig. 5A–C). Animals treated with GAL-1 also showed an increase in the levels of the anti-inflammatory cytokine IL-10 (Fig. 5D) compared to the levels in animals treated with IR.

*In vitro*, HR induced an increase in MCP-1 levels in HK-2 cell supernatants at 4, 16 and 24 h (Fig. 5F), IL-1β levels at 16 h (Fig. 5E) and IL-8 levels at 8 and 16 h (Fig. 5G), as well as reductions in IL-10 levels at 16 h (Fig. 5H). Pre-treatments with GAL-1 and DEXA abolished these effects on the cytokine release in HK-2 cells. The addition of exogenous β-galactose did not alter the anti-inflammatory activity of GAL-1 in the production of these cytokines.

### HR on HK-2 cells modulates GAL-1 endogenous expression and release

**Figure 6:**
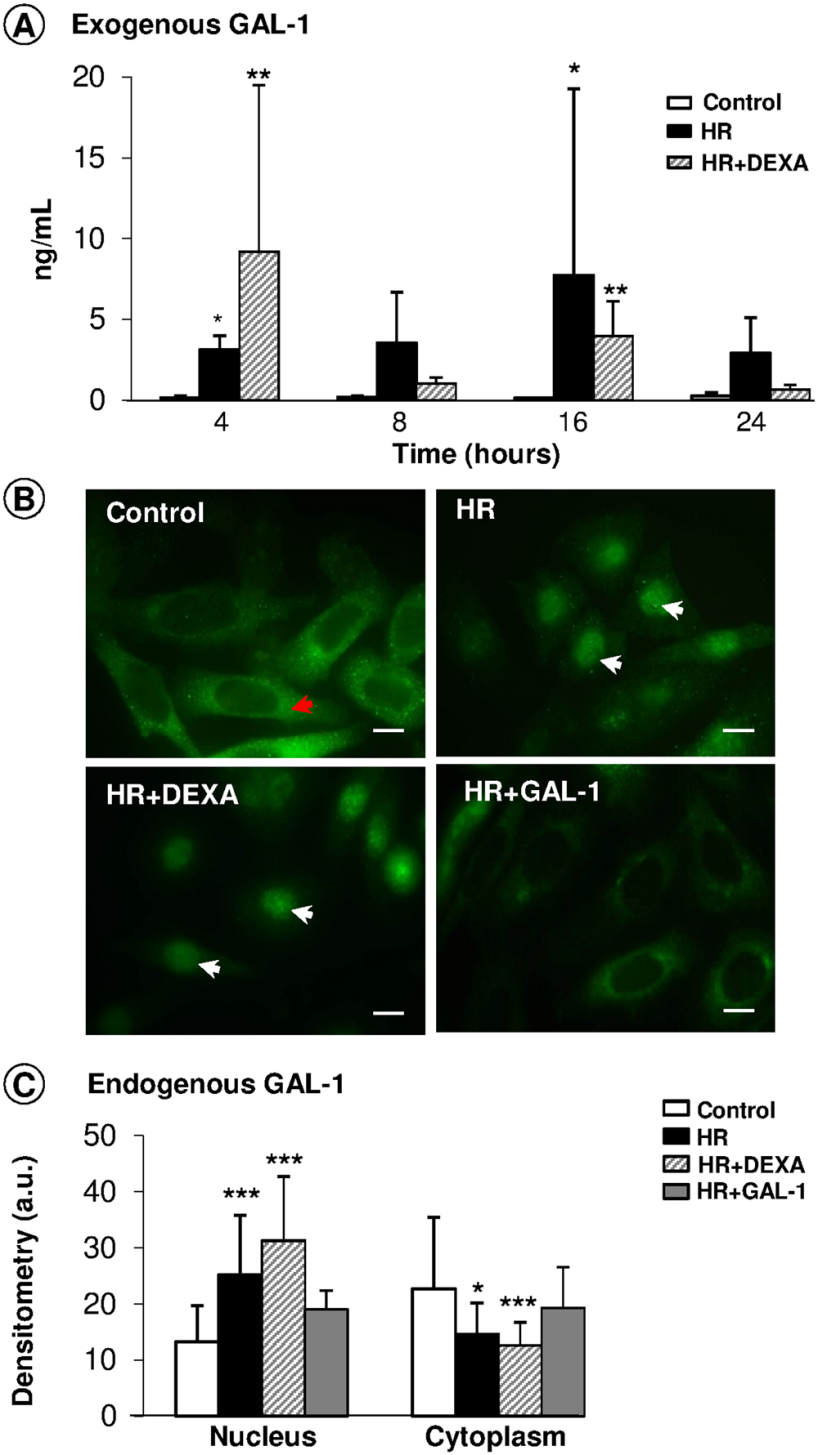
Expression of endogenous and exogenous GAL-1 in the HK-2 cell assays. **(A)** HR and DEXA induced GAL-1 release in the supernatants of cells at 4 and 16 h. Data are expressed as the mean ± SE (ng/mL), N = 3/group. *p < 0.05 and **p < 0.01, vs control. (**B)** GAL-1 expression in the cytoplasm (red arrows) and in the nucleus (white arrows) of the HK-2 cells under different experimental conditions. Bars: 10 μm. (**C)** Densitometric analysis of GAL-1 expression in the HK-2 cells. Data (arbitrary units, a.u.) represent the mean ± SD of GAL-1 immunoreactivity. *p < 0.05, **p < 0.01 and ***p < 0.001, vs control, Kruskal-Wallis test.

The release of GAL-1 was investigated in the supernatants of HK-2 cells under control, HR and DEXA-treated HR conditions at different time points. Increased levels of GAL-1 were exhibited in the HR condition at 4 and 16 h, as well as in treatment with DEXA, compared with control cells (Fig. 6A).

In accordance with these results and those obtained with cytokine release in HK-2 cell supernatants, the time course of 16 h was chosen for the analysis of endogenous GAL-1 expression through immunofluorescence. These studies were conducted in control, HR and DEXA or GAL-1-treated HR conditions (Fig. 6B). Control HK-2 cells exhibited GAL-1 expression predominantly in the cytoplasm, and after the HR assay, higher GAL-1 levels in the nucleus were evidenced. This pattern of endogenous GAL-1 immunostaining was reversed by GAL-1 treatment but not by DEXA. Densitometry confirmed these observations, showing that GAL-1 expression was increased in the nucleus and diminished in the cytoplasm of HK-2 cells after HR, treated or not treated with DEXA, compared to the expression in other groups (Fig. 6C). No significant alterations in the GAL-1 immunoreactivity were detected between control/HR and GAL-1-treated cells.

## Discussion

IR-induced renal injury results from a complex process involving the release of reactive oxygen species and proinflammatory mediators, the upregulation of adhesion molecules and leukocyte recruitment that culminates with rapid kidney dysfunction and high mortality rates^2,4^. Here, an experimental *in vivo* rat model of renal IR and an *in vitro* HR model with a human proximal tubule cell line were used. We tested GAL-1 as a suitable therapeutic tool to diminish the side effects of IR-induced renal injury.

The IR procedure used in our study is widely employed^3,16–18^, and right nephrectomy followed by left IR is a model designed with the objective of creating an underlying decrease of 50% of nephron mass and rat survival of at least a few days to study acute kidney injury. Considering that no polyuria and hyperfiltration was detected in the IR group at the 48 h time point, instead of reduced urine output and creatinine clearance, hypertrophy of the remaining kidney did not occur in our study.

Initially, we verified that treatment with GAL-1 30 min before the IR process, as well as DEXA, reversed the damage on renal function, as it reduced plasma creatinine and BUN and sodium/potassium excretion and increased the creatinine clearance and urine volume. *In vitro* studies confirmed this protective effect of GAL-1 and DEXA on tubular cells (HK-2 cells), which rescued their viability after the HR assay.

Our histological findings revealed no differences in the score of tubular necrosis between untreated or treated (GAL-1 or DEX) IR groups. Despite this, the untreated IR group exhibited elevated urine levels of KIM-1, a transmembrane glycoprotein of renal tubules that is considered a sensitive and specific marker for renal proximal injury monitoring^19–22^, an effect mitigated by GAL-1 treatment but not by DEXA. Our results are in accordance with a previous study using the IR model that also showed no differences in the KIM-1 urinary levels after 24 and 120 h of DEXA treatment compared to the untreated group^23^. We emphasize that KIM-1, undetectable in normal kidneys, has consistently been demonstrated as a biological marker of early renal damage^21,22,24^, which indicates a partial protective effect of GAL-1 in our study.

Corroborating the KIM-1 data and kidney injury, we found an elevation in cell death by apoptosis in IR animals, which was reversed by GAL-1 treatment but not by DEXA. In fact, the anti-apoptotic role of GAL-1 is possibly related to the activation of focal adhesion kinase-1 (FAK1) in hypoxic epithelial cells^25^. In this study, GAL-1 treatment increased the phosphorylation of FAK1, whereas inhibition of GAL-1 reduced hypoxia-induced pFAK1 levels in H441 cells (human lung adenocarcinoma cells). Furthermore, GAL-1 inhibition activated caspase-3 in the lungs of mice injured with bleomycin and hypoxia, indicating that GAL-1 inhibition can induce the apoptotic pathway in hyperactivated epithelial cells via reduced FAK1 activation.

Similar protective effects of GAL-1 on renal function have been observed in a rat model of glomerulopathy^26^. Alternate-day therapy with GAL-1 (i.p. administration; 1 mg/kg) during two weeks of reduced proteinuria and renal macrophage influx was induced by rabbit nephrotoxic serum. However, GAL-1 treatment failed to inhibit apoptosis and the influx of CD8^+^ cells and to reduce the anti-rabbit IgG production. In contrast, the injection of GAL-1 into allogeneic rat renal recipients can significantly prolong animal survival and decrease the CD8^+^ T cell subsets in a dose-dependent manner^27^. These studies suggest that the therapeutic effect of GAL-1 may be mediated by several different mechanisms, including the modulation of cell–cell or cell–matrix adhesion and proliferation.

In fact, the anti-migratory effect of GAL-1 on leukocytes has been associated with the expression of L-selectin and ß2-integrin^13–15,28,29^ and described in different experimental models of inflammation^7,9,30^. As expected, in our study, the intravenous administration of GAL-1 or DEXA was also sufficient to reduce macrophage and neutrophil influx into the renal interstitium of the juxtamedullary region under the IR condition, corroborated by diminished recruitment of double-positive L-selectin/β2-integrin blood leukocytes, in comparison with that in the untreated IR group.

The anti-migratory effect of GAL-1 on macrophages and neutrophils in the IR-injured kidneys probably resulted from the decreased expression of ICAM-1 in the juxtamedullary region. Accordingly, ICAM-1-null mice or wild-type mice treated with ICAM-1 antibody are protected from renal IR injury^30–32^. Intratubular leukocytes are known to adhere to ICAM-1-positive tubular cells in the renal biopsies of patients with progressive glomerulonephritis^33^, and post-ischaemic kidneys express increased levels of adhesion molecules on endothelial cells^34^. Therefore, increased expression of ICAM-1 contributes to leukocyte recruitment and infiltration into post-ischaemic tissue and then leads to enhanced leukocyte-interactions, which can promote kidney injury^2,34^.

Increased influx of leukocytes to tissue contributes to the enhancement of reactive oxygen species (ROS), which causes oxidative stress^35–37^. In addition, ROS and peroxynitrite generation contributes to nitrotyrosine formation, a marker of nitrosative stress^36–38^. Here we also demonstrated that IR induced elevated plasma TBARS and nitrotyrosine renal levels. GAL-1 treatment partly abolished these alterations once plasma TBARS levels were not reduced. Collectively, these data suggest an antioxidant protective effect of the GAL-1 protein through regulation of macrophage/neutrophil influx to the renal tissue.

In the next step of our study, we examined the expression of endogenous GAL-1 under the IR condition. The rat kidneys exhibited GAL-1 immunoreactivity in the cortical region, mainly in the glomeruli and walls of the blood vessels, confirming previous findings^39–41^. After IR, increased GAL-1 expression was detected in the interstitial tissue of necrosed tubules of the juxtamedullary region. Another study using a rat model of IR injury revealed an increased number of GAL-1-positive interstitial cells in the vicinity of altered tubules after 48 h, which declined at one week to reach control values^41^. GAL-1 overexpression was also detected in rodent kidneys with Meckel syndrome renal cystic and juvenile cystic kidney diseases relative to the normal kidney^42,43^. Additionally, the increased number of GAL-1-positive cells in patients with diffuse mesangial proliferation and glomerulosclerosis in comparison with control patients indicates that GAL-1 may be crucially involved in the pathogenesis of glomerulopathy in these subjects^39^. However, glomeruli from human nephritic syndrome had a significantly lower expression of GAL-1 than the control glomeruli from individuals with minor glomerular abnormalities. These observations were corroborated by a rat model of puromycin aminonucleoside-induced nephrosis that showed drastically reduced GAL-1 expression in the glomeruli at days 2 and 5^40^. Such differences in GAL-1 expression in kidney diseases reflect the pleiotropic functions of this lectin in renal tissues, which will be further evaluated to address innovative therapies.

*In vitro*, after the HR process, HK-2 cells markedly increased the nuclear expression of GAL-1 and decreased its cytoplasmic levels relative to control cells, which suggests nuclear protein translocation. Similar data have been observed in a tubular HKC cell line submitted to a high-glucose condition and TGF-β1 treatment^41^. Equally important, the HR condition augmented GAL-1 levels in HK-2 cell supernatants, detected at 4 and 16 h, showing this cellular type as a potential source of this lectin, as corroborated by previous data^39,44^.

Exogenous treatment with GAL-1 decreased its endogenous expression in the juxtamedullary interstitium in relation to the IR group and abrogated its high nuclear levels under the HR condition. Similar results were observed in rodent models of endotoxin-induced uveitis and allergic conjunctivitis^28,29^, where the inflammatory response increases the expression of endogenous GAL-1 in eye tissues, while treatment with exogenous GAL-1 reduces its endogenous expression, possibly through a process of inhibitory self-regulation. On the other hand, in a rat acute rejection model of allogeneic renal transplantation, injection of GAL-1 increased its endogenous expression in the recipient rat renal cortex in a dose-dependent fashion, but the exact mechanism of the increased GAL-1 expression was unclear. Furthermore, GAL-1 therapy plays an important role in inducing allogeneic rat immunotolerance after renal transplantation^27^.

Finally, to better understand the role of GAL-1 in the IR condition, we analysed the urine of animals and supernatants of HK-2 cells under different conditions. Again, GAL-1 and DEXA treatments exhibited an anti-inflammatory function *in vivo* by decreased urine levels of IL-1β, MCP-1, and TNF-α and increased IL-10 and *in vitro* by reducing the release of IL-1β, MCP-1 and IL-8 in an independent carbohydrate-recognition-domain manner (with addition of β-galactose). These data corroborate previous experimental models of acute inflammatory or autoimmune diseases in which exogenous GAL-1 reduces the levels of TNF-α, IL-1β, IL-2, IL-6, IL-8, IL-12 and IFN-γ and increases IL-5 and IL-10, consequently regulating the activity of leukocytes^6,7,9,13,27,45–48^.

Despite the anti-inflammatory, antioxidant and antiapoptotic effects of GAL-1 on kidney IR model, no protective effect was evidenced in the acute tubular necrosis, an unexpected finding. A possible explanation could be the multifactorial aspect of renal injury models. In fact, studies involving protective handling on renal injury models have shown controversial results. The administration of hydroxytyrosol, a natural antioxidant of olive oil, completely counteracts cyclosporine A (CsA)-induced oxidative stress, the decreased lipid peroxidation level and haem oxygenase-1 (HO-1) gene expression in rats. However, the renal histology is partially protected^49^. Similarly, pharmacological treatment with annexin A1, a glucocorticoid-induced and anti-inflammatory protein, partially protected against CsA-induced histological changes in an acute CsA nephrotoxicity model^50^. On the other hand, the blockade of the renin-angiotensin system by the administration of losartan and enalapril prevents fibrosis induced by CsA, but does not improve renal function^51^. Thus, future studies using a chronic IR model will be necessary to address and confirm the protective effect of GAL-1 on the development of fibrosis, as well as in cytokine/chemokine release and kidney blood flow.

In summary, the results showed the pleiotropic protective effects of GAL-1 in the early phase of IR-induced kidney injury by improving renal function, inhibiting the release of proinflammatory mediators and KIM-1, and downregulating the expression of the adhesion molecule, which culminates in reduced leukocyte transmigration. Our data indicate that GAL-1 is a new biological target for therapeutic intervention in inflammatory kidney conditions.

## Methods

### Ethics statement

This study was approved and carried out in accordance with the recommendations of the Ethics Committee on Animal Experimentation of São Paulo State University, UNESP, which approved the protocol (N. 055/2011).

### ***In vivo*** study: renal IR injury

Male Wistar rats (200–250 g) were randomly distributed into the following four groups (*N* = 6/group): SHAM (control), IR, IR treated with GAL-1 or dexamethasone (DEXA). The animals were housed under a 12 h:12 h light–dark cycle and allowed access to food and water ad libitum.

The IR procedure consisted of right nephrectomy and occlusion of the left renal artery with a non-traumatic vascular clamp for 30 min^16,17^. This model was selected because it causes moderate to severe, reversible renal injury in a consistent and reproducible way^16,17^. Recombinant human GAL-1 (100 μg/animal; PeproTech, London, UK) or DEXA (100 μg/animal; Sigma-Aldrich, St. Louis, MO, USA) were administered intravenously 30 min before surgery; the untreated IR group received only phosphate-buffered saline (PBS). The doses of GAL-1 and DEXA were selected according to previous studies^26,27^ and after preliminary experiments at our laboratory. Animals from the SHAM group were submitted to the same anaesthesia (20 and 50 mg/mL of xylazine and ketamine, respectively - 1 mL/100 g rat) and surgical procedures described above but without the renal artery clamped. The animals were euthanized 48 h after reperfusion.

### Renal function study

After 1 day of IR or SHAM procedure, the rats were placed in metabolic cages, with urine volume collected and measured for 24 h, and samples were taken at the end of this period. Furthermore, the animals were euthanized through an anaesthesia overdose, blood samples were drawn, and renal tissue samples were collected. Sodium and potassium levels were determined using an electrolyte analyser, and creatinine levels were ascertained with a colorimetric assay in the 24-h urine and in the blood samples. The blood urea nitrogen (BUN, urea/2.1428) was also ascertained with a colorimetric assay. Commercial kits were used for the measurements of creatinine and urea (Biotecnica, Varginha, MG, Brazil).

### Determination of plasma thiobarbituric-acid-reactive substances (TBARS)

Plasma TBARS was determined by quantification of substances that react with thiobarbituric acid, generating colouring that might be detected in a spectrophotometer at 535 nm, compared to the reading of the absorbance of the standard malondialdehyde 20 µM/L^52,53^. The measurement of TBARS is a well-established method for monitoring lipid peroxidation.

### Histopathology

The kidneys were fixed in a 4% paraformaldehyde in PBS 0.1 M (pH 7.4) for 24 h at 4 °C and embedded in paraffin for histopathology and immunohistochemistry. Acute tubular necrosis was evaluated in the juxtamedullary region stained with haematoxylin-eosin, and the scores were assigned as follows: 0 (no field affected), 1 (up to 25% affected), 2 (26 to 50% affected), 3 (51 to 75% affected) and 4 (76 to 100% affected), as previously described^16^. Quantitative analysis of neutrophils was performed in tissue sections from the juxtamedullary segments in a blinded fashion, with counting being performed using a high-power objective (63×) on an Axioskop 2-Mot Plus Zeiss microscope (Carl Zeiss, Jena, Germany). The number of neutrophils was quantified, and the values are reported as the mean (±SD) number of cells/mm^2^.

### Immunohistochemistry

Caspase-3, nitrotyrosine, macrophages (ED-1, CD68 positive cells), GAL-1 and ICAM-1 (intercellular adhesion molecule-1) staining was performed in 3 µm sections of paraffin-embedded kidneys. After an antigen-retrieval step using citrate buffer pH 6.0, the endogenous peroxide activity was blocked, and the sections were incubated overnight at 4 °C with a primary rabbit polyclonal anti-caspase-3 Ab (1:1000, 9662, Cell Signaling Technology, Danvers, MA, USA), anti-GAL-1 Ab (1:200, Zymed Laboratories, Cambridge, UK) or mouse monoclonal anti-ICAM-1 Ab (1:100, NB500–318, Novus Biological, Littleton, USA). For macrophage and nitrotyrosine detection, the sections were incubated for 1 h at room temperature with primary mouse monoclonal anti-CD68 Ab (1:500, MCA341R, Serotec, Oxford, UK) or anti-nitrotyrosine Ab (1:400, SC-32757, Santa Cruz Biotechnology, CA, USA). After washing, the sections were incubated with a secondary biotinylated antibody (LAB-SA Detection kit, Invitrogen, Paisley, UK). Positive staining was detected using a peroxidase-conjugated streptavidin complex, and colour was developed using DAB substrate (Invitrogen). The sections were counterstained with haematoxylin.

Quantitative counting of macrophages was performed as described for neutrophils, and the values are reported as the mean (±SEM) number of cells/field. Densitometry for caspase-3, nitrotyrosine, GAL-1 and ICAM-1 was performed in 25 fields of the juxtamedullary region from each animal (n = 6 per group). The values are shown as the mean ± SEM of arbitrary units (a.u.) using AxioVision software (Carl Zeiss, Jena, Germany).

### Blood flow cytometry

To quantify L-selectin (CD62L) and β2-integrin (CD11b) positive cells under different experimental conditions, we collected whole blood in EDTA from an abdominal cava vein puncture (n = 6 per group). Aliquots (10 µL) of animal blood were incubated with 1 μL of a monoclonal antibody against L-selectin conjugated with phycoerythrin (PE; anti-rat CD62L; BD PharMingen, San Diego, CA) and β2-integrin conjugated with fluorescein isothiocyanate (FITC; anti-rat CD11b; BD PharMingen) diluted 1:10 in PBS for 20 min at 4 °C in the dark. Immediately after incubation, 180 µL of Guava Lysing Solution/Fixative (Millipore Corporation, USA) was added to lyse and fix the cells for 20 min at 37 °C. The cells were analysed using a Guava easyCyte flow cytometer (Millipore Corporation, USA), and leukocytes were gated according to the side and forward scatter parameters. Data were obtained from 10,000 cells (only morphologically viable cells were considered in the analysis) to determine the percentages of CD62L- and CD11b-positive cells.

### ***In vitro*** study model: hypoxia/reoxygenation injury

HK-2 cells (CRL-2190, human proximal tubule cell line derived from normal kidney; American Type Culture Collection) were grown following the manufacturer’s instructions. The medium was replaced by DMEM:F-12 without phenol supplemented with 10% FBS, 200 mM L-glutamine, 0.1 mg/mL streptomycin, and 100 U/mL penicillin (Invitrogen, Frederick, MD, USA) and incubated for 24 h. After the equilibration period, HK-2 cells were divided into two atmospheric conditions: oxygenated (control) and hypoxia-reoxygenation (HR) pre-treated or not pre-treated with GAL-1 or DEXA (3 independent experiments/group). In control cells, pO_2_ was maintained within the 200–300-mmHg range. Hypoxia (pO_2_: 20–40 mmHg) was induced by superfusing the cell atmosphere with 95% N_2_/5% CO_2_ for 5 min. After 30 min of hypoxia, the cells were re-oxygenated with 95% O_2_/5% CO_2_ for 5 min (pO_2_: 200–300-mmHg) and maintained under this condition for 45 min^17,54,55^. Recombinant GAL-1 (4 μg/mL; PeproTech; alone or plus β-galactose 100 mM; (Sigma-Aldrich), to inhibit the carbohydrate recognition domain^28^, or DEXA (1 µM; Sigma-Aldrich), as a positive control for anti-inflammatory therapy, was added to the media 30 min before the HR assay. At 4, 8, 16 and 24 h after reoxygenation, the cells and supernatants were collected for the following assays.

The cytotoxic effect of the HR condition was determined by the trypan blue dye exclusion assay. The HK-2 cells were trypsinized, and an equal volume of cell suspension was mixed with trypan blue. Viable cells were counted as clear cells, and dead cells, as blue ones using a Countess Automated Cell Counter (Invitrogen, Frederick, MD, USA). The number of live cells per millilitre was calculated using the following formula:

$$ \% {\rm{viability}}=({\rm{live}}\,{\rm{cell}}\,{\rm{count}}/{\rm{total}}\,{\rm{cell}}\,{\rm{count}})\times 100.$$

### Immunofluorescence

To detect the expression of GAL-1, we grew HK-2 cells on coverslips for 16 h after HR, fixed them in 4% paraformaldehyde for 24 h, washed them in PBS, Tween 20 (0.4%), blocked them with 1% BSA diluted in 3% normal goat serum, and incubated them with polyclonal rabbit anti-GAL-1 Ab (42-5900, Invitrogen, Camarillo, USA; 1:100 in normal goat serum 1.5%). After washing, the cells were incubated with a goat anti-rabbit Ab conjugated with FITC (Serotec, Oxford, UK; 1:100 in normal goat serum 1.5%) for 1 h. The slides were mounted with a solution containing glycerol and PBS (1:1). Negative control samples were incubated only with normal goat serum without primary Ab. The cells were analysed using a filter with a wavelength of 546 nm on an Axioskop 2-Mot Plus Zeiss microscope, and the levels of the protein were quantified by densitometry.

### Analysis of chemical mediators in the urine of IR-animals and the supernatants of HR-HK-2 cells

For urine cytokine tests, 25 µL of sample was employed (n = 6 per group), using the MILLIPLEX RAT Cytokine/Chemokine Magnetic Bead panel (RECYTMAG-65K; Millipore Corporation, USA), following the manufacturer’s instructions. The kit can detect MCP-1, IL-1β, IL-10 and TNF-α cytokines simultaneously in a single sample and examines them using Luminex laser-based fluorescent analytical test instrumentation (MAGPIX, Austin, TX). Cytokine concentrations were determined from standard curves prepared on each plate and expressed in picograms per millilitre (pg/ml).

KIM-1 (kidney injury molecule 1) levels in urine and IL-1β, IL-8, IL-10, MCP-1 and GAL-1 levels in the HK-2 cell supernatants were performed with commercially available immunoassay kits (R&D Systems, Minneapolis, MN, USA), also according to the manufacturer’s instructions. The cytokine concentration of each sample was calculated from the standard curve.

### Statistical analysis

After normality determination by Kolmogorov–Smirnov test, data with a normal distribution were subjected to analysis of variance (ANOVA), followed by the Newman–Keuls post hoc test for multiple comparisons. Samples with a non-normal distribution were subjected to the Kruskal–Wallis test followed by Dunn’s test. A *p* value of <0.05 was considered significant.
